# Effects of Fermented Feed on the Growth Performance, Intestinal Function, and Microbiota of Piglets Weaned at Different Age

**DOI:** 10.3389/fvets.2022.841762

**Published:** 2022-04-08

**Authors:** Shuai Liu, Hao Xiao, Yunxia Xiong, Jingping Chen, Qiwen Wu, Xiaolu Wen, Zongyong Jiang, Li Wang

**Affiliations:** State Key Laboratory of Livestock and Poultry Breeding, Ministry of Agriculture Key Laboratory of Animal Nutrition and Feed Science in South China, Guangdong Key Laboratory of Animal Breeding and Nutrition, Maoming Branch, Guangdong Laboratory for Lingnan Modern Agriculture, Institute of Animal Science, Guangdong Academy of Agricultural Sciences, Guangzhou, China

**Keywords:** fermented diet, weaning age, intestinal function, intestinal microbiota, tight junction

## Abstract

The beneficial function of fermented feed in livestock industry has been widely investigated. However, little is known about the effects of fermented feed on different weaned-day piglets. This study aimed to investigate the effects of fermented diet on the growth performance, intestinal function, and microbiota of piglets weaned at the age of 21 and 28 days. The results found that weaning on day 21 significantly increased (*p* < 0.05) average daily gain (ADG), and average daily feed intake (ADFI) (calculated based on wet weight and dry matter), while reduced (*p* < 0.05) feed to gain ratio (F:G), the activities of trypsin and lipase of jejunum and the villus height of ileum, compared with 28-days weaning. The protein levels of Occludin, Claudin-1, and ZO-1 of ileum in the groups weaning on day 21 were less (*p* < 0.05) than the groups weaning on day 28. Moreover, dietary supplementation with fermented diet upregulated (*p* < 0.05) the Occludin, Claudin-1, and ZO-1 proteins of ileum, compared with the groups treated with control diet both weaning on day 21 and 28. In addition, dietary supplementation with fermented diet decreased (*p* < 0.05) the relative abundance of *Clostridia* (class) and increased (*p* < 0.05) the *Bacteroidia* (class) level of cecal microbiota, compared with the groups treated with control diet both weaning on day 21 and 28. However, supplementation with fermented diet did not affect the concentrations of short-chain fatty acids in the cecum (*p* > 0.05). Therefore, our data suggest that the feed digestibility is improved in piglets weaned at 21 days, but intestinal barrier function is weaker than in piglets weaned at 28 days. However, compared with feeding control diet, supplementation with fermented diet both improved the feed conversion and intestinal barrier function of weaned piglets by modulating intestinal microbiota.

## Introduction

Weaning would harm the intestinal function of piglets and lead to food intake reduction, diarrhea risk increasing, and death in severe cases (1, 2). Weaning affects the production performance. Jamil et al. found that the average daily gain (ADG) and average daily feed intake (ADFI) of piglets increased linearly with the increasing weaning age (3). Pigs weaned at 14 days old grow slower, and the feed intake are reduced compared with those weaned at 24 days old (3). Compared with weaning on 12 days, weaning on 20 days increased ADG (4). Alison has pointed out that delaying weaning can improve feed digestibility (5). Another study showed that the villi height of jejunum in weaned piglets was significantly lower than unweaned piglets at the same age (6, 7). According to Blecha et al. (8), early weaning can cause passive immunity weakened in piglets. The study of Janeen found that piglets weaned at 28 days of age were stronger in immunity and had more antibodies after exposure to the virus than piglets weaned at 14 days of age (9). Weaning age affects the performance, immunity, and intestinal function of piglet. Therefore, weaning age is an extremely important parameter that affects the growth of piglets.

Soybean meal is widely used in pig production as a plant-derived protein source. However, soybean is limited to piglets' diet because of the anti-nutritional factors, such as soybean globulin, β-glycoprotein, trypsin inhibitor, and non-starch polysaccharide, which can exacerbate weaning stress, increase diarrhea risk, and hinder the growth rate of piglets. Currently, the use of microbial fermented feed is a common method to reduce antinutritional factors in feed. The amount of soybean antigenic proteins (β-conglycinin and glycinin) in feed was decreased after fermented by *Bacillus subtilis* (10). Feeding fermented feed can improve the intestinal health of piglets, increase productivity, and change the intestinal microbial structure (11). A study has shown that the mixed solid-state fermentation of *B. subtilis, Hansenula anomala*, and *Lactobacillus casei* can improve the nutrient digestion of piglets (12). Another study showed that fermented feed can increase the concentration of butyric acid in the cecum of piglets (13). On the other hand, fermented diet can provide probiotics and their metabolites, which improve the digestibility of feed and the intestinal function of weaned piglets (14–16).

However, studies on the effects of fermented feed on piglets at different weaning days are still lacking. Therefore, the present study was conducted to investigate the effects of fermented feed, weaning age, and their interaction effect on the growth performance, intestinal function, and microbiota of piglets, and to provide a new insight for pig production.

## Materials and Methods

All animal procedures used in the present study were approved by the Animal Care and Use Committee of Guangdong Academy of Agricultural Sciences and followed the Guidelines for the Care and Use of Animals for Research and Teaching (Authorization number GAASIAS-2016-017).

### Fermented Feed Preparation

*Bacillus subtilis, Saccharomyces cerevisiae*, and *Lactobacillus* (*L. casei* and *Lactobacillus plantarum*) were used to ferment feed materials (corn, soybean meal, and soybean hull) at room temperature in this study. A mixture of *B. subtilis* and *S. cerevisiae* is considered as bacteria A that was purchased from Prosyn (Haerbing, China), and the number of viable *B. subtilis* is 7.2 × 10^9^ CFU/g, the number of viable *S. cerevisiae* is 3.1 × 10^9^ CFU/g. *Lactobacillus* is considered as bacteria B that was purchased from Inner Mongolia Sci-Plus Biotech (Inner Mongolia, China), and the total amount of viable *L. casei* + *L. plantarum* is 5.1 × 10^9^ CFU/g. Bacteria A and B were mixed at a ratio of 1:1 as fermentation strains. Fermented feed was prepared with 18.1 kg ingredients, 6.9 kg water, and 25 g fermentation strains, and then fermented in the laboratory for 5 days. The ingredients used to ferment were mixed at a ratio completely corresponding with the basal diet (Table 1). The contents of anti-nutritional factors in the materials before and after fermentation are shown in Table 2. Fermented feed was blended with other ingredients before used as fermented diet.

**Table 1:** Ingredients and nutrient levels of basal diet (air-dry basis).

**Ingredients**	**Content, %**	**Nutrient levels^**b**^**	
Corn	32.92	ME^c^, kcal/kg	3403.00
Expanded corn	15.00	CP^d^, %	19.00
Soybean meal	10.00	Ca, %	0.76
Soybean hulls	5.00	Total P, %	0.76
Extruded soybean	12.00	AP^e^, %	0.53
Soybean oil	1.50	SID^f^ Lys, %	1.35
Fish meal	6.50	SID^f^ Met+Cys, %	0.74
Whey powder	11.00	SID^f^ Thr, %	0.79
Lactose	1.00	SID^f^ Trp, %	0.22
Sucrose	1.00	SID^f^ Ile, %	0.63
Choline chloride (50%)	0.30	SID^f^ Val, %	0.70
Salt	0.45	SID^f^ Arg, %	0.97
Calcium hydrogen phosphate	0.70	SID^f^ His, %	0.39
Calcium citrate	0.50	SID^f^ Leu, %	1.24
Phytase	0.02	SID^f^ Phe, %	0.69
L-Lys HCl	0.60		
DL-Met	0.23		
L-Thr	0.23		
L-Trp	0.05		
Premix^a^	1.00		
Total	100.00		

*^a^Provided per kilogram of diet: 12,400 IU vitamin A, 2,800 IU vitamin D3, 30 mg vitamin E, 5 mg vitamin K3, 3 mg thiamin, 10 mg riboflavin, 40 mg niacin, 8 mg pyridoxine, 40 μg vitamin B12, 0.08 mg biotin, 15 mg pantothenic acid, 1 mg folic acid, 80 mg Zn, 120 mg Fe, 70 mg Mn, 16 mg Cu, 0.7 mg I, 0.3 mg Se. ^b^Values were calculated according to NRC (17). ^c^ME, metabolic energy. ^d^CP, crude protein. ^e^AP, available phosphorous. ^f^SID, standardized ileal digestible*.

**Table 2:** Contents of anti-nutritional factors (mg/g).

**Items**	**Ingredients before fermented**	**Ingredients after fermented**	**Basal diet**	**Fermented diet**
β-Conglycinin	166.51	67.57	108.31	51.02
Glycinin	233.66	63.45	129.69	44.14
Trypsin inhibitor factor	1.34	0.28	0.51	0.18

### Animals and Experimental Design

**Figure 1:**
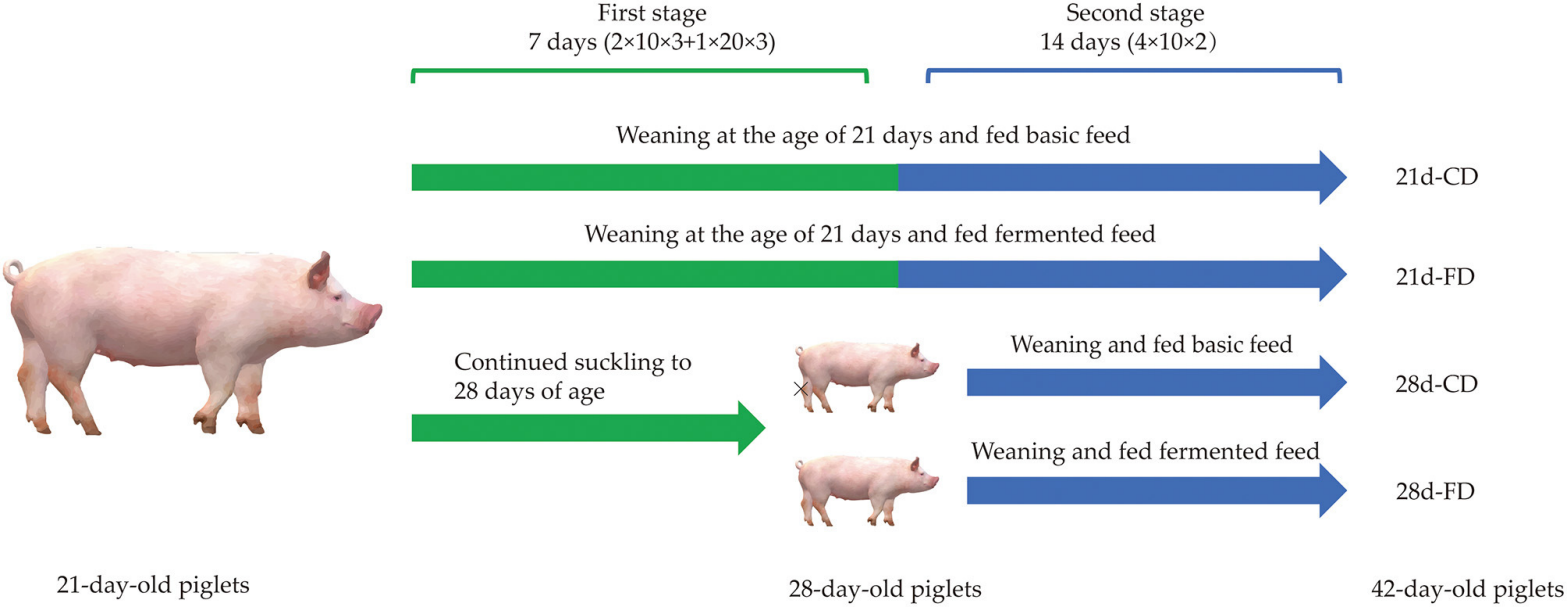
Diagram of the experimental design. Six 21-day-old piglets with similar body weight were randomly selected from the same litter of 20 sows, and then three of them were randomly selected as a replicate and to be weaned immediately, and the others continue to suckle until 28 days old before weaning. As soon as weaned, the piglets were randomly allocated into two treatments, fed with basal diet as the control group and fermented diet as the fermented group, respectively. The experiment was finished at the age of 42 days old. 21d-CD, 21-day-old weaned piglets fed the control diet. 21d-FD, 21-day-old weaned piglets fed the fermented diet. 28d-CD, 28-day-old weaned piglets fed the control diet. 28d-FD, 28-day-old weaned piglet fed the fermented diet. The same as below.

As shown in Figure 1, a 2 × 2 two-factor design was adopted in this study. In this study, six 21-day-old piglets [Duroc × Landrace × Yorkshire, body weight (BW) about 6.15 ± 0.02 kg] were selected from the same litter of 20 sows, and 3 among them were randomly selected as a replicate and to be weaned, and the others were continued to suckle until 28 days old before weaning. As soon as the piglets were weaned, the replicates were randomly allocated into two treatments, fed with basal diet as the control group or fermented diet as the fermented group, respectively. The feed trial was finished when the piglets were at the age of 42 days old. Piglets with approximate average BW were randomly selected from each replicate to slaughter and sample at the age of 28 and 42 days old, respectively. The basal diet (Table 1) was formulated according to NRC (17) to meet the nutritional requirements of piglets about 7–11 kg (17) without high copper and zinc supplementation. The nutrient composition of the experiment diet before and after fermentation is shown in Table 3. During the whole experiment, the animals were free to access feed and water. Once the experiment was finished, ADG, ADFI, and feed to gain ratio (F:G) were calculated.

**Table 3:** Nutrient levels in before and after fermentation.

**Items**	**Before fermented**	**After fermented**
Water, %	10.83	27.96
CP^a^, %	18.53	19.68
GE^b^, MJ/kg	16.86	17.18
EE^c^, %	3.74	2.49
CF^d^, %	4.41	3.44
NDF^e^, %	4.53	2.45
ADF^f^, %	3.29	1.75
Ash, %	5.56	5.18
Calcium, %	1.69	1.63
Phosphorus, %	0.57	0.52

*^a^CP, crude protein. ^b^GE, gross energy. ^c^EE, ether extract. ^d^CF, crude fiber. ^e^NDF, neutral detergent fiber. ^f^ADF, acid detergent fiber. The nutrient levels were determined value based on dry matter*.

### Sample Collection

At the end of the experiment, after fasting overnight, all animals were sacrificed after intramuscular (i.m.) injection with sodium pentobarbital (40 mg/kg BW). The digesta from the stomach, jejunum, ileum, cecum, and colon were carefully collected and snap frozen in liquid nitrogen. Tissue samples from the proximal duodenum, middle jejunum, and distal ileum were dissected, and one sample was immediately frozen in liquid nitrogen for subsequent protein expression analysis, and another sample was fixed with 4% paraformaldehyde for subsequent morphological analysis.

### Measurement of Digestive Enzyme Activity

The activities of trypsin, lipase, and amylase were determined by commercial kits from the Nanjing Jiancheng Bioengineering Institute (Nanjing, China). Homogenize the sample with the sample dilution at a ratio of 1:10. The homogenate was centrifuged at 4,500 rpm for 10 min at 4°C. The supernatant was taken for the enzymatic assay. The protein concentrations of sample were determined using a BCA kit (Thermo Fisher, USA), and the final results were expressed as per milligram protein.

### Intestinal Histopathological Examination

The intestinal morphology was examined according to the method of Gao et al. (18). Briefly, after paraffin embedded fixed, intestinal segments were cut into 5-μm sections. Then, the sections were dewaxed with xylene, hydrated with alcohol, and stained with hematoxylin and eosin (H&E). Images were obtained by using a fluorescent orthochromatic microscope (Haier, China). In total, 10 bright fields were randomly selected for each section. The villus height, and crypt depth were measured using image-pro image processing software (Media Cybernetics, Rockville, MD).

### Western Blotting

Furthermore, 100 mg of the sample was taken in an RNAse-free tube with 1 ml of protein lysis buffer (Biosharp, Anhui, China) on ice for 30 min, then centrifuged at 12,000 rpm and the supernatant was taken. After measuring the supernatant protein concentration by the BCA kit (Thermo Fisher, USA), then the protein lysate diluted the supernatant to the same concentration. The diluted sample was mixed with the loading buffer (Beyotime, Shanghai, China) at a ratio of 1:5 and denatured at 100°C for 10 min. Then, the proteins were separated by 10% sodium dodecyl sulfate–polyacrylamide gel electrophoresis (SDS-PAGE), and transferred onto a nitrocellulose membrane (BIO-RAD, USA). After blocking with blocking buffer (Beyotime, Shanghai, China), the membranes were incubated with the corresponding primary antibody at 4°C for 12–16 h, followed by incubation with horseradish peroxidase (HRP)-conjugated secondary antibody for 1 h. Bands were detected using the ECL PlusTM Western Blotting Substrate (CliNX, Shanghai, China). Band intensity was quantified using ImageJ software (ImageJ 1.52, National Institutes of Health, USA). Primary antibodies for β-actin (1:10,000), zonula occludens-1 (ZO-1, 1:250), Occludin (1:1,000), and Claudin-1 (1:1,000) (Abcam, MA, USA) were used in this study.

### Microbiota Profiling

The total genomic DNA of cecal content samples was extracted by using the OMEGA Soil DNA Kit (Omega Bio-Tek, Norcross, GA, USA, #D5625-01) according to the manufacturer's instructions and stored at −20°C. A nanodrop ND-1000 spectrophotometer (Thermo Fisher Scientific, Waltham, MA, USA) was used to quantify the sample DNA, and the DNA quality was checked by 1.2% agarose gel electrophoresis. The PCR amplification of bacterial 16S rRNA genes V3-V4 regions was performed using forward primer 338F (5′-ACTCCTACGGGAGGCAGCA-3′) and the reverse primer 806R (5′-TCGGACTACHVGGGTWTCTAAT-3′). Sample-specific 7-bp barcodes were incorporated into the primers for multiplex sequencing. The PCR components contained 5 μl buffer (5 ×), 0.25 μl Fast pfu DNA Polymerase (5 U/μl), 2 μl dNTPs (2.5 mM), 1 μl forward and reverse primer (10 μM), 1 μl DNA template, and 14.75 μl ddH_2_O. The PCR amplification program consisted of initial denaturation at 98°C for 5 min, followed by 25 cycles of denaturation at 98°C for 30 s, annealing at 53°C for 30 s, elongation at 72°C for 45 s, and then a final extension at 72°C for 5 min. PCR products were recovered and quantified by Vazyme VAHTSTM DNA Clean Beads (V azyme, Nanjing, China) and the Quant-iT PicoGreen dsDNA Assay Kit (Invitrogen, Carlsbad, CA, USA), respectively. Paired-end 2 × 250 bp sequencing was performed using the Illumina kit and the Illumina MiSeq platform (Shanghai, China).

The bioinformatic analysis was conducted with QIIME2 pipeline. Briefly, the clean reads were obtained by de-primed, quality filtered, denoised, spliced, and de-chimerism using a DADA2 method from raw reads (19). After merged into amplicon sequence variants (ASVs) feature sequence, singletons ASVs removed, the ASVs were clustered using QIIME2's classify-sklearn algorithm (20) and then annotation using Naive Bayes classifier based on Greengenes database (21). The Rarefaction method is used to normalize and classify the ASVs with a depth of 95% (22, 23). Chao1 (24) index and Observed species index were used to characterize richness, and Shannon (25) index and Simpson (26) index were used to characterize the evenness of the bacterial community. Principal component analysis (PCA) based on ASVs physical distance, and principal coordinate analysis (PCoA) based on unweighted Unifrac distance are used to display the distribution of samples.

### Determination of Short-Chain Fatty Acids

Short-chain fatty acids in the cecal content were determined according to the method of Xiong et al. (27). Briefly, the samples of cecum contents were added to NaOH and caproic acid-d3, and centrifuged to obtain supernatant. The supernatant was mixed with water, propyl/pyrimidine, and propyl chloroformate, and to derive for 5 min. Finally, the supernatant was extracted two times with hexane, and the final supernatant was dehydrated with Na_2_SO_4_. The determination was carried out with a gas chromatograph-mass spectrometer detector (7890A and 5975C inert XL EI/CI mass spectrometric detector, Agilent Technologies, Santa Clara, CA).

### Statistical Analysis

The SPSS 25 software (IBM, USA) was used for statistical analysis. The effects of fermented diet, weaning age, and their interaction effect were assessed by ANOVA using a general linear model (GLM) procedure. Then, we checked for the homogeneity of variance assumption by performing the Levene test (*p* > 0.05). Results are represented as means and pooled standard error (SE), and *p* < 0.05 indicates significant differences.

## Results

### Weaning Days and Fermented Diet Both Affect the Growth Performance of Piglets

Body weight, ADG, ADFI (calculated based on wet weight and dry matter), and F:G are shown in Table 4. The BW on day 21 and 28 were similar among the treatments. Dietary supplementation with fermented diet increased (*p* < 0.05) BW on day 42, compared with the groups treated with control diet both weaning on day 21 and 28. Weaning on day 21 significantly increased (*p* < 0.05) ADG, and ADFI (calculated based on wet weight and dry matter), and reduced F:G, compared with 28-day weaning. Piglets fed fermented diet had higher (*p* < 0.05) ADFI (calculated based on wet weight) during 28–42 days old, but F:G was lower (*p* < 0.05), compared with piglets fed with the control diet. There was no interaction effect between fermented diet and weaning age (*p* > 0.05) on all indexes.

**Table 4:** The effects of fermented diet and weaning age on the growth performance of piglets.

**Items**	**21-d**		**28-d**		**SEM**	***P*** **-value**		
	**CD**	**FD**	**CD**	**FD**		**F**	**W**	F*W
BW at 21 days old, kg	6.17	6.15		6.15				
BW at 28 days old, kg	7.01	7.07	8.71	8.80	1.05	0.338	0.125	0.102
BW at 42 days old, kg	11.54	12.21	11.69	12.36	0.91	0.021	0.603	0.997
ADG, g/d	323.6	363.25	212.04	230.09	73.61	0.009	<0.001	0.900
^a^ADFI, g/d	488.76	592.28	345.71	426.16	101.41	<0.001	<0.001	0.494
^b^ADFI, g/d	444.34	422.88	314.28	304.28	75.96	0.259	<0.001	0.679
F: G	1.45	1.19	1.59	1.31	0.24	<0.001	0.041	0.872

21-d, weaning at 21 days old. 28-d, weaning at 28 days old. CD, the control diet. FD, the fermented diet. BW, body weight. ADG, average daily gain. ADFI, average daily feed intake. F: G, feed: gain ratio. F, the main effect of fermented diet. W, the main effect of weaning age. F * W, the interaction effect between fermented diet and weaning age. Data are expressed as mean and pooled SEM. ^a^ADFI was calculated based on weight; ^b^ADFI was calculated based on dry matter.

### Weaning Days and Fermented Diet Both Affect Digestive Enzyme Activity

**Figure 2:**
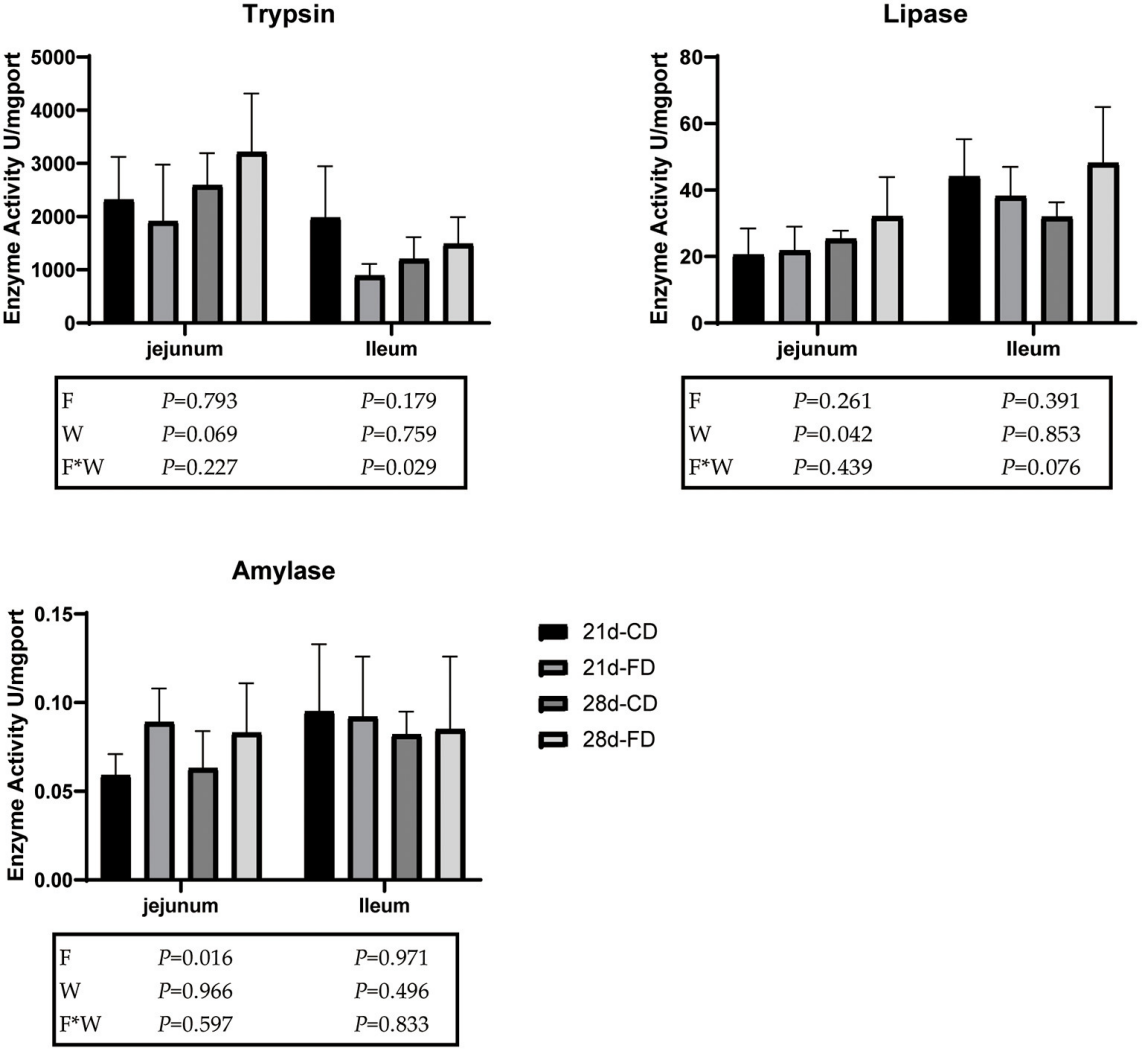
The effects of fermented diet and weaning age on digestive enzyme activity. 21d-CD, 21-day-old weaned piglets fed the control diet. 21d-FD, 21-day-old weaned piglets fed the fermented diet. 28d-CD, 28-day-old weaned piglets fed the control diet. 28d-FD, 28-day-old weaned piglet fed the fermented diet. F, the main effect of fermented diet. W, the main effect of weaning age. F*W, the interaction effect between fermented diet and weaning age. Values are presented as the mean ± SEM.

The trypsin activity, lipase activity, and amylase activity of jejunum and ileum are shown in Figure 2. Compared with the groups treated with control diet both weaning on day 21 and 28, the dietary supplementation with fermented diet had no significant effect on the lipase activity of ileum (*p* > 0.05). However, there was interaction effect between fermented diet and weaning age (*p* < 0.05) on the lipase activity and amylase activity of ileum. There was no difference (*p* > 0.05) in the trypsin activity and lipase activity of the jejunum between the groups treated with control diet and the groups supplemented with fermented diet among weaning on day 21 and 28. Compared with 28-days weaning, weaning on day 21 significantly decreased (*p* < 0.05) the trypsin activity and lipase activity of jejunum. Dietary supplementation with fermented diet increased (*p* < 0.05) the amylase activity of jejunum, compared with the groups treated with control diet both weaning on day 21 and 28. There was no interaction effect between fermented diet and weaning age (*p* > 0.05) on the trypsin activity, lipase activity, and amylase activity of jejunum.

### Weaning Days and Fermented Diet Have no Significant Effect on Intestinal Morphology

**Figure 3:**
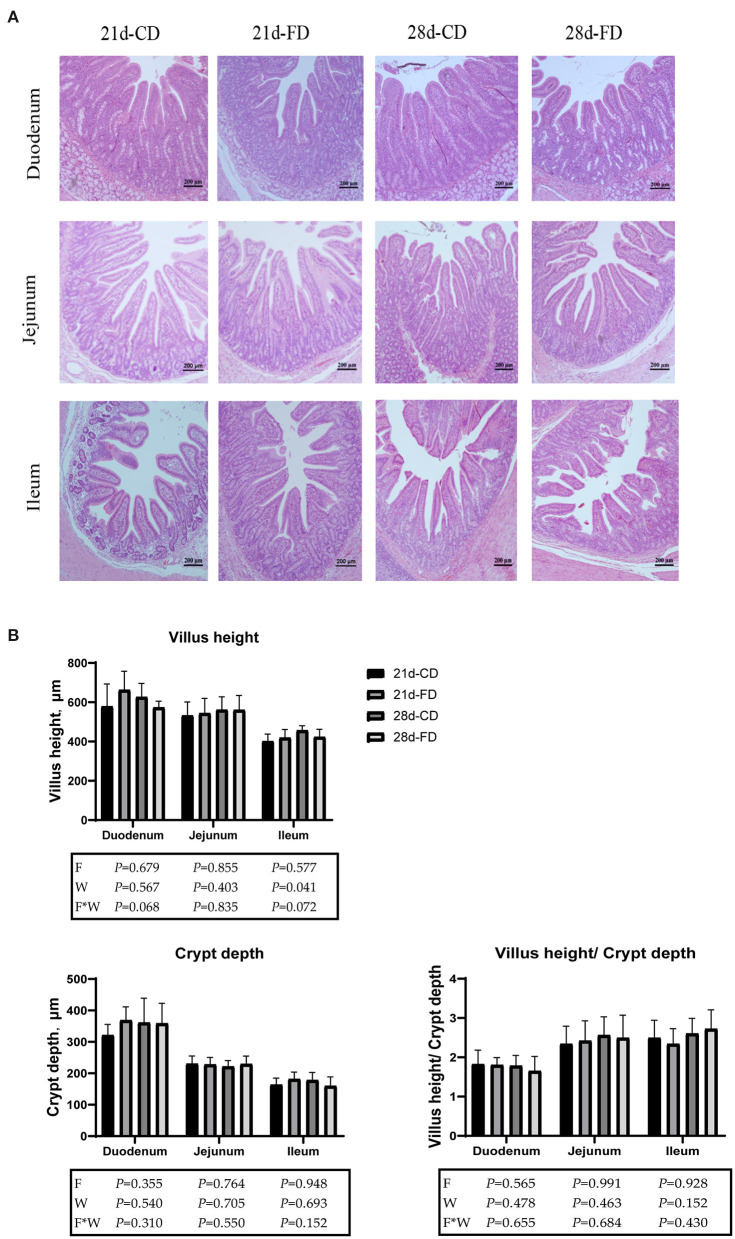
Hematoxylin-eosin staining photos of the duodenum, jejunum, and ileum (magnification × 200). **(A)** The duodenum, jejunum, and ileum by H&E-staining (scale bar 200 μm). **(B)** Villi height, crypt depth and villi height/crypt depth in duodenum, jejunum and ileum. 21d-CD, 21-day-old weaned piglets fed the control diet. 21d-FD, 21-day-old weaned piglets fed the fermented diet. 28d-CD, 28-day-old weaned piglets fed the control diet. 28d-FD, 28-day-old weaned piglet fed the fermented diet. F, the main effect of fermented diet. W, the main effect of weaning age. F*W, the interaction effect between fermented diet and weaning age. Values are presented as the mean ± SEM.

Sections of duodenum, jejunum, and ileum in each group are shown in Figure 3A. The morphology of intestinal villi was normal in all groups. Villus height, crypt depth, and villus height/crypt depth are shown in Figure 3B. The villus height, crypt depth, and villus height/crypt of duodenum and jejunum were similar (*p* > 0.05) among the treatments. Compared with 28-days weaning, weaning on day 21 significantly reduced (*p* < 0.05) villus height of ileum. However, weaning days did not affect (*p* > 0.05) the crypt depth and villus height/crypt depth of ileum. Dietary supplementation with fermented diet did not affect (*p* > 0.05), compared with the groups treated with control diet both weaning on day 21 and 28. There was no interaction effect between fermented diet and weaning age (*p* > 0.05) on all indexes.

### Weaning Days and Fermented Diet Both Affect the Expression of Tight Junction Proteins

**Figure 4:**
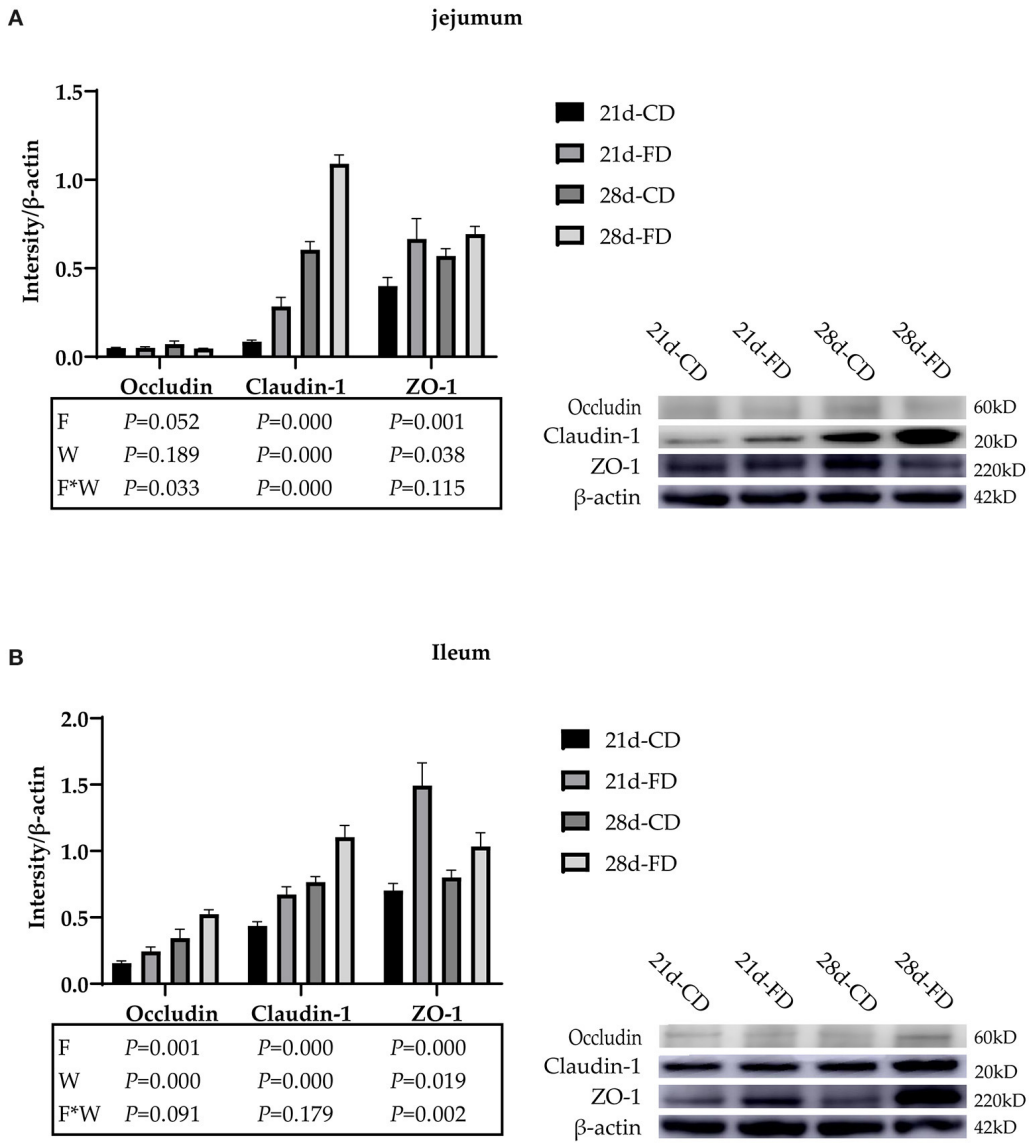
The effects of fermented diet and weaning age on the tight junction proteins expression in the small intestine of weaned piglets. The protein expression of Occludin, Claudin, and ZO-1 in the jejunum **(A)** and ileum **(B)** were detected *via* western blotting, and the intensity of the bands was determined using Image J. 21d-CD, 21-day-old weaned piglets fed the control diet. 21d-FD, 21-day-old weaned piglets fed the fermented diet. 28d-CD, 28-day-old weaned piglets fed the control diet. 28d-FD, 28-day-old weaned piglet fed the fermented diet. F, the main effect of fermented diet. W, the main effect of weaning age. F*W, the interaction effect between fermented diet and weaning age. Values are presented as the mean ± SEM.

The relative expression of jejunal tight binding protein is shown in Figure 4A. Although the Occludin proteins of jejunum among the treatments was not affected (*p* > 0.05) on day 21 and 28, it is affected (*p* < 0.05) by the interaction of fermented diet and weaning age. Weaning on day 21 significantly downregulated (*p* < 0.05) the Claudin-1 proteins of jejunum, compared with 28-days weaning. Dietary supplementation with fermented diet upregulated (*p* < 0.05) the Claudin-1 proteins of jejunum, compared with the groups treated with control diet both weaning on day 21 and 28. There was interaction effect between fermented diet and weaning age (*p* > 0.05) on the Claudin-1 proteins of jejunum. The effect of weaning age and fermented diet on ZO-1 proteins is similar (*p* > 0.05) to Claudin-1 proteins, but there is no interaction on ZO-1 proteins. The relative expression of ileum tight binding protein is shown in Figure 4B. Weaning on day 21 significantly downregulated (*p* < 0.05) the Occludin, Claudin-1, and ZO-1 proteins of ileum, compared with 28-days weaning. Dietary supplementation with fermented diet upregulated (*p* < 0.05) the Occludin, Claudin-1, and ZO-1 proteins of ileum, compared with the groups treated with control diet both weaning on day 21 and 28. There was no interaction effect between fermented diet and weaning age (*p* > 0.05) on Occludin and Claudin-1. There was interaction effect between fermented diet and weaning age (*p* < 0.05) on ZO-1 proteins.

### Weaning Days and Fermented Diet Both Affect the Richness of Microbiota

**Figure 5:**
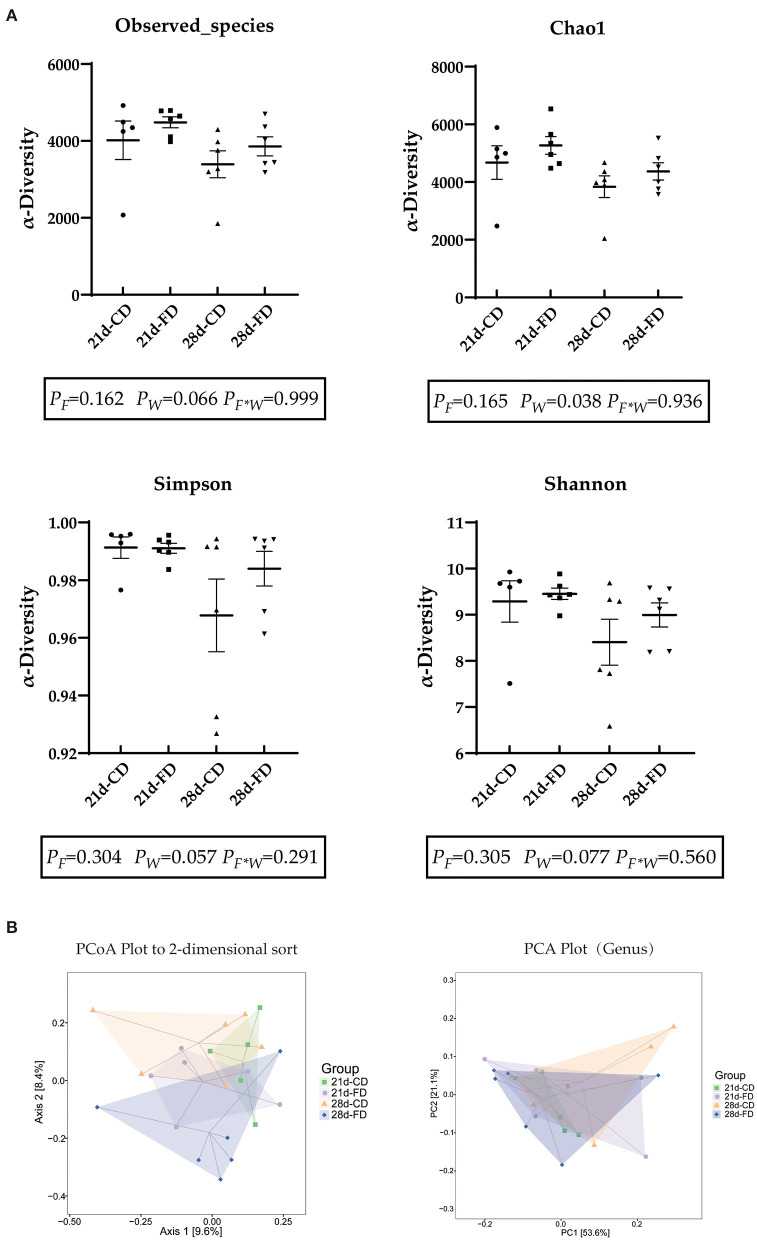
The effects of fermented diet and weaning age on the cecal microbial composition of piglets. **(A)** Observed-species, Chao1 index, simpson index and Shannon index were used to measure α-diversity; **(B)** Unweighted Unifrac distance-based PCoA plot and OTUsbased PCA plot (genus). 21d-CD, 21-day-old weaned piglets fed the control diet. 21d-FD, 21-day-old weaned piglets fed the fermented diet. 28d-CD, 28-day-old weaned piglets fed the control diet. 28d-FD, 28-day-old weaned piglet fed the fermented diet. F, the main effect of fermented diet. W, the main effect of weaning age. F*W, the interaction effect between fermented diet and weaning age. Values are presented as the mean ± SEM.

In this experiment, a total of 25 cecal content samples were collected for 16S rRNA sequencing. After removing low-quality sequences, 139,985 clean reads were clustered into 4,281 ASVs. The complexity of species diversity was estimated by diversity indexes (Shannon and Simpson) and richness indexes (observed species and Chao1). As shown in Figure 5A, weaning days had no significant influence (*p* > 0.05) on the Observed species index, Chao1 index, Simpson index, and Shannon index among the treatments. The Observed species index, Simpson index, and Shannon index of dietary supplementation with fermented diet were similar (*p* > 0.05), compared with the groups treated with control diet both weaning on day 21 and 28. Weaning on d 21 significantly reduced (*p* < 0.05) Chao-1 index compared with 28-days weaning. There was no interaction effect between fermented diet and weaning age (*p* > 0.05) on all indexes. As displayed in Figure 5B, the PCA and PCoA plot indicated that both weaning age and fermented diet had an effect on the gut microbial structure of piglets.

### Weaning Days and Fermented Diet Both Affect Intestinal Microbial Structure

**Figure 6:**
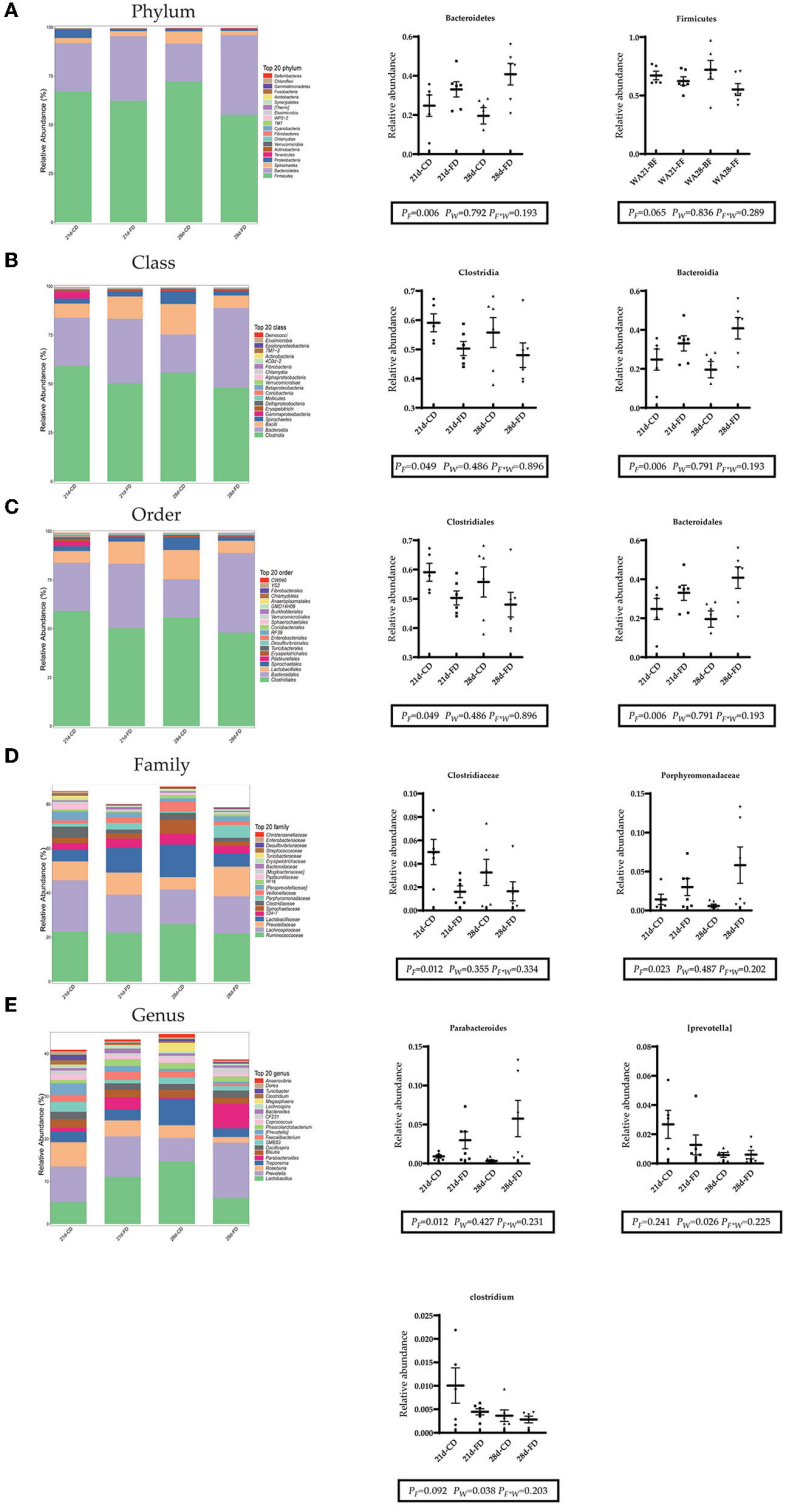
Composition of the top 20 bacteria at the level of phylum, family, and genus. **(A–E)** The left side of the figure shows the composition of the top 20 bacteria at each level, and the right side shows the top 20 differential bacteria at each level. 21d-CD, 21-day-old weaned piglets fed the control diet. 21d-FD, 21-day-old weaned piglets fed the fermented diet. 28d-CD, 28-day-old weaned piglets fed the control diet. 28d-FD, 28-day-old weaned piglet fed the fermented diet. F, the main effect of fermented diet. W, the main effect of weaning age. F*W, the interaction effect between fermented diet and weaning age. Values are presented as the mean ± SEM.

The top 20 bacteria at levels of phylum, class, order, family, and genus were selected for analysis. As shown in Figure 6A at the phylum level, *Bacteroidetes* (phylum) and *Firmicutes* (phylum) were dominant in the gut which accounted for more than 90%. Weaning days had no significant effect (*p* > 0.05) on the abundance of *Bacteroidetes* (phylum) and *Firmicutes* (phylum) among the treatments. Dietary supplementation with fermented diet increased (*p* < 0.05) the abundance of *Bacteroidetes* (phylum), compared with the groups treated with control diet both weaning on day 21 and 28. The abundance of *Firmicutes* (phylum) was similar (*p* > 0.05) between dietary supplementation with fermented diet and the groups treated with control diet both weaning on day 21 and 28. As shown in Figures 6B,C at the class level and order level, both weaning on day 21 and 28 did not affect (*p* > 0.05) on the abundance of *Clostridia* (class), *Bacteroidia* (class), *Clostridiales* (order), and *Bacteroidales* (order). Dietary supplementation with fermented diet increased (*p* < 0.05) the abundance of *Bacteroidia* (class) and *Bacteroidales* (order), reduced (*p* < 0.05) the abundance of *Clostridia* (class) and *Clostridiales* (order), compared with the groups treated with control diet both weaning on day 21 and 28. As shown in Figure 6D at the family level, both weaning on day 21 and 28 did not affect (*p* > 0.05) on the abundance of *Clostridiaceae* (family) and *Porphyromonadaceae* (family). Compared with the groups treated with control diet both weaning on day 21 and 28, dietary supplementation with fermented diet reduced (*p* < 0.05) the abundance of *Clostridiaceae* (family), and increased (*p* < 0.05) *Porphyromonadaceae* (family). As shown in Figure 6E at the genes level, both weaning on day 21 and 28 did not affect (*p* > 0.05) on the abundance of *Parabacteroides* (genus). Dietary supplementation with fermented diet increased (*p* < 0.05) the abundance of *Parabacteroides* (genus), compared with the groups treated with control diet both weaning on day 21 and 28. Weaning on day 21 significantly increased (*p* < 0.05) the abundance of *[prevotella]* (genus) and *Clostridium* (genus), compared with 28-days weaning. Dietary supplementation with fermented diet did not affect (*p* > 0.05) the abundance of *Parabacteroides* (genus) and *Clostridium* (genus), compared with the groups treated with control diet both weaning on day 21 and 28. There was no interaction effect between fermented diet and weaning age (*p* > 0.05) on all indexes.

### Weaning Days and Fermented Diet Do Not Affect the Short-Chain Fatty Acids of Cecum

The short-chain fatty acid of cecal contents is shown in Table 5. Weaning day had no significant effect (*p* > 0.05) on the content of acetic acid, propionic acid, butyric acid, valeric acid, isobutyric acid, and isovaleric acid in cecum among the treatments. Dietary supplementation with fermented diet did not affect (*p* < 0.05) the content of acetic acid, propionic acid, butyric acid, valeric acid, isobutyric acid, and Isovaleric acid in cecum, compared with the groups treated with control diet both weaning on day 21 and 28. There was no interaction effect between fermented diet and weaning age (*p* > 0.05) on all indexes.

**Table 5:** The effects of fermented diet and weaning age on the concentrations of short-chain fatty acids in cecal content of piglets (mg/kg).

**Items**	**21-d**		**28-d**		**SEM**	***P*** **-value**		
	**CD**	**FD**	**CD**	**FD**		**F**	**W**	F*W
Acetic acid	1.83	7.21	3.36	3.97	1.37	0.298	0.763	0.405
Propionic acid	597.20	799.68	688.84	654.92	39.00	0.286	0.733	0.140
Butyric acid	273.34	396.92	310.20	308.38	26.01	0.255	0.624	0.241
Valeric Acid	56.98	93.36	76.82	85.30	8.70	0.218	0.742	0.438
Isobutyric acid	22.39	34.99	168.12	28.95	23.52	0.153	0.116	0.900
Isovaleric acid	29.03	39.46	31.46	36.08	2.74	0.194	0.933	0.609

21-d, weaning at 21 days old. 28-d, weaning at 28 days old. CD, the control diet. FD, the fermented diet. F, the main effect of fermented diet. W, the main effect of weaning age. F^*^W, the interaction effect between fermented diet and weaning age. Data are expressed as mean and pooled SEM.

## Discussion

Studies have shown that fermented feed can improve the growth performance of weaned piglets (12, 28, 29). Fermented feed has attracted much attention in recent pieces of research, whereas the weaning age was often neglected. Few studies have focused on the interaction effect between fermented feed and weaning age. The present study was conducted to investigate the effects of fermented feed on the growth performance, intestinal function, and microbiota of piglets weaned at different age.

In production, piglets are often weaned at 21 days of age. Faccin et al. (30) found that the production performance of piglet improved linearly with increasing weaning days. A study by Dinan (31) showed that whether the piglets weaned at 21 days old or weaned at 28 days old, there was no significant difference in BW at 46 days old. The retardation of weaning age increases ADG in short term, but the increasing in BW is offset within 14 days post weaning (31). Although the 28-d weaned piglets had a higher ADG during the period from 21 to 28 days old, the ADG and ADFI during 28 to 42 days old decreased, and F:G increased. This implies that compared with 21-d weaning, postponing the weaning age to 28 days old, the feed intake reduction caused by the weaning stress has not been effectively relieved. The results of a previous study showed that whether piglets weaned at 14, 21, or 28 days old, there was no significant difference in BW when they reached 42 days old (32).

In this study, supplementation with fermented feed increased the ADG during 28–42 days of age, and the results are consistent with previous studies (33, 34). Compared with the control diet group, the fermented diet group increased the ADFI, and decreased F/G. After fermentation, the macromolecular substances in corn and soybean meal are decomposed into small molecular substances that are more conducive to the absorption for piglets, and improves the utilization rate of feed. A study has shown that the β-sheet structure in *B. subtilis* fermented soybean meal is reduced by 43.2%, and the more easily absorbed random coil structure is increased by 49.9% (35).

Few literatures have studied on the effects of fermented feed on the digestive enzyme activity of weaned piglets. Fed with soybean meal fermented by *B. subtilis* was reported to increase the total protease and trypsin in the duodenum and jejunum of piglets (36). Inconsistently, in this study, feeding the fermented diet had no effect on the digestive enzymes in the stomach, jejunum, and ileum. Different weaning age may be one main reason to explain the different results. In this paper, the feed trial was lasted for 22 days, and the test time may be too short for fermented feed to change the digestive tract enzyme activity of piglets. Differences in the fermentation bacterial strains and fermentation processes may be another reason. A study by Lei (32) has shown that as weaning age increases, the rate of fat absorption by piglets also increases. In this study, the jejunal lipase activity at 42 days old of 28-days weaned piglets was significantly higher than that of 21-days weaned piglets. The lipase activity increased along with the retardation of weaning age, and the digestion and absorption of fat promoted.

In this study, 28-days weaning significantly increased the ileal villus height, but had no significant effect on the villus height/crypt depth. The small intestinal morphology of 21-days weaned piglets was damaged due to the weaning stress, and manifested in the decreasing of intestinal villi height and crypt depth deeper. A study has shown that the small intestinal villus in 28-days weaned piglets shrink to the shortest on the 3rd day after weaning, and begins to recover on the 5th day (32). Another study showed that the small intestine of piglets weaned at the age older than 28 days old recovered faster when suffering weaning stress (6). Weaning age affect the recovery speed of the small intestine suffering weaning stress, but on the 14th day post weaning, the villus height of the small intestine can be restored to the length before weaning (1, 37), and that is also consistent with the results of our study. However, the fermented diet had no significant effect on the integrity of the small intestine. Moreover, the piglets used in this study are self-breeding and self-raising on our farm, with strict biological prevention and control, and transportation stress greatly reduced.

Tight junction proteins, such as Claudins, ZO-1, and Occludin are important components of the intestinal barrier (38), which selectively permeate nutrients and water, and block pathogens (39, 40). Extracellular regulated protein kinases (ERK1/2) activation of ETS-like 1 transcription factor 1 (Elk-1) can lead to the upregulation of pro-inflammatory cytokines interferon gamma (IFN-γ) and tumor necrosis factor (TNF-α), which in turn damage the intestinal barrier functions and increase intestinal permeability (41–43). Early weaning stress can lead to the activation of mitogen-activated protein kinase (MAPK) signaling pathway ERK1/2, resulting in an inflammatory response in the intestine. A study has shown that 14 days post weaning, the relative mRNA expression of Occludin, Claudin-1, and ZO-1 in the jejunal mucosa was dropped (1), intestinal permeability was augmented, and the intestinal barrier functions were weakened. In this study, compared with piglets fed control diet, supplementation with fermented diet significantly increased the relative expression of Occludin, Claudin-1, and ZO-1 in the small intestine. The results indicates that fermented diet can improve the intestinal barrier functions of piglets. As for the weaning age, the intestinal barrier functions of 28-days weaned piglets is stronger, and there is a certain interaction effect between feed diet and weaning age. Another study showed that toll-like receptor 2 (TLR 2) enhances the expression of ZO-1 to maintain the integrity of the intestinal barrier by protein kinase C (44), and the TLR recognition may be affected by the regulation of intestinal microbiota (45). In this study, both fermented diet and 28-days weaning improved the damage of the intestinal barrier functions, but the specific effect of that pathway needs to be further explored.

Crosstalk exists between the intestinal microbiota and the host. Commensal microbes that inhabit the gut participate in the digestion and absorption of nutrients, mediation of immunity system, and take part in the host's metabolism *via* their metabolites. In turn, the alterations of intestinal microbiota reflect the state of the host (46, 47). Weaning stress causes the volatility of gut microbiota in piglets (48, 49). A study has shown that *Fusobacterium (*genus), *Akkermansia (*genus)*, Clostridiales (*genus), *Deltaproteobacteria (*genus), and *Selenomonadales (*genus) in the intestine of post-weaned diarrheal piglets are increased compared with healthy weaned piglets, while *Prevotella (*genus), and *Faecalibacterium (*genus) decreased (50). *Bacteroidales (*order) and *Clostridiales (*order) are the two most dominant orders involved in glycolysis in the large intestine (51, 52). In this study, fermented diet reduced the relative abundance of *Clostridiales (*order) and increased the relative abundance of *Bacteroidales (*order). Fermented diet increased the relative abundance of beneficial bacteria, thereby improving the intestinal barrier functions. Fermentation of insoluble polysaccharide by microbiota in the hindgut yields short-chain fatty acids, which take part in improving the intestinal health of weaned piglets (53). However, in this study, the composition of the intestinal microbiota has been changed, and the alterations in short-chain fatty acids producing bacteria can also be found, but the contents of short-chain fatty acids in the intestine did not change significantly.

## Conclusions

In summary, delaying the weaning time can improve the feed conversion and intestinal barrier function. Supplementation with fermented diet significantly reduces F:G during 28–42 days of age in piglets, and improves the small intestinal barrier functions and cecal microbial structure. Supplementation with fermented diet improve the feed efficiency and intestinal barrier functions of weaned piglets by modulating intestinal microbiota. Moreover, an addition of fermented feed has a significant effect on the intestinal barrier of piglets at different weaning ages. Thus, both delayed weaning and the use of fermented feeds can be beneficial for piglets, but in production, the costs and benefits need to be further evaluated.

## Data Availability Statement

The datasets presented in this study can be found in online repositories. The names of the repository/repositories and accession number(s) can be found at: https://www.ncbi.nlm.nih.gov/, https://www.ncbi.nlm.nih.gov/sra/PRJNA779532.

## Ethics Statement

The animal study was reviewed and approved by Guangdong Academy of Agricultural Sciences and followed the Guidelines. Written informed consent was obtained from the owners for the participation of their animals in this study.

## Author Contributions

SL, YX, and JC performed experiments, analyzed data, and wrote the manuscript. QW and XW performed experiments. HX, ZJ, and LW supervised the project, developed the study concept, and wrote and edited the manuscript. All authors read and approved the final manuscript.

## Funding

This study was supported by the China Agriculture Research System of MOF and MARA; Special fund for the scientific innovation strategy-construction of high level Academy of Agriculture Science (R2018PY-JC001, R2020PY-JG009, R2017YJ-YB1004, and R2018PY-QF001); the Project of Swine Innovation Team in Guangdong Modern Agricultural Research System (2021KJ126); and Independent Research and Development Projects of Maoming Laboratory (2021ZZ003).

## Conflict of Interest

The authors declare that the research was conducted in the absence of any commercial or financial relationships that could be construed as a potential conflict of interest.

## Publisher's Note

All claims expressed in this article are solely those of the authors and do not necessarily represent those of their affiliated organizations, or those of the publisher, the editors and the reviewers. Any product that may be evaluated in this article, or claim that may be made by its manufacturer, is not guaranteed or endorsed by the publisher.

## Abbreviations

BW: Body weight
ADG: Average daily gain
ADFI: Average daily feed intake
F:G: Feed to gain ratio
ZO-1: Zonula occluden-1
ERK1/2: Extracellular regulated protein kinases
Elk-1: ETS-like 1 transcription factor 1
IFN-γ: Interferon gamma
TNF-α: Tumor necrosis factor
MAPK: Mitogen-activated protein kinase
TLR 2: Toll-like receptor 2.
